# The association between ultra-processed food intake and age-related hearing loss: a cross-sectional study

**DOI:** 10.1186/s12877-024-04935-0

**Published:** 2024-05-23

**Authors:** Yanpeng Fu, Wenyu Chen, Yuehui Liu

**Affiliations:** 1 Department of Otorhinolaryngology Head and Neck Surgery, Second Affiliated Hospital of Nanchang University, Nanchang, China; 2 Interventional Cardiology Department, Second Affiliated Hospital of Nanchang University, No. 1 Minde Road, Nanchang, China

**Keywords:** Hearing loss, Ultra-processed foods, Age-related, NHANES, Cross-sectional survey

## Abstract

**Objectives:**

This study aimed to explore the association between ultra-processed foods and age-related hearing loss.

**Methods:**

Cross-sectional analyses based on data from a nationally representative sample of 1075 adults aged over 50 in the US was performed. The odds ratios (ORs) and 95% confidence intervals (CIs) for hearing loss according to ultra-processed foods intake quartiles were calculated using a multiple adjusted logistic regression model. Restricted cubic spline model was used to flexibly model potential nonlinear relations between ultra-processed foods intake and possibility of hearing loss. We also explored statistical interactions and conducted subgroup analyses where they were found to be significant.

**Results:**

Ultra-processed foods intake was significantly correlated with high-frequency hearing loss. After controlling for all covariables, individuals in the fourth quartile of Ultra-processed foods consumption had a 2.8 times higher chance of developing high-frequency hearing loss than individuals in the first quartile of Ultra-processed foods consumption. We also found that the association was more significant in non-Hispanic whites.

**Conclusions:**

This study discovered an association between Ultra-processed foods intake and the incidence of high-frequency hearing loss, which was more significant in non-Hispanic whites.

**Supplementary Information:**

The online version contains supplementary material available at 10.1186/s12877-024-04935-0.

## Introduction

Age-related hearing loss (ARHL) is a condition in which the auditory system deteriorates with age. The WHO estimated that roughly 25% of those over 60 have been impacted by debilitating hearing loss in 2021 [1] and there will be more than 500 million people effected by ARHL worldwide in 2025 [2]. ARHL is most noticeable at higher frequencies and usually develops after age of 50 [3]. Hearing loss not only has an impact on the physical and psychological health [4, 5], but it also places a huge social and economic burden on families and society [6]. As a result, ARHL is a huge burden from both a public health and a social standpoint.

Association between ARHL and nutrients or broader dietary patterns have been explored in previous studies. The Nurses’ Health Study II found that eating a diet rich in fruits, vegetables, nuts, chicken, and fish, along with moderate alcohol consumption, reduced the risk of hearing loss [7]. Huang et al. [8] found that adherence to a Mediterranean-style diet was negatively correlated with ARHL in people from the US.

The term “ultra-processed foods” (UPF) refers to industrial formulations made from ingredients taken from foods, usually with the addition of flavors, colors, and other cosmetic additions, according to the NOVA food categorization system. Ultra-processed foods account for more than half of all dietary energy in high-income countries and one fifth to one third of total dietary energy in middle-income countries [9]. In high-income countries, sales of UPFs rise at around 1% per year, whereas in middle-income countries, sales expand at up to 10% per year [10]. Several epidemiological studies have evaluated the association of ultra-processed foods with chronic diseases, including cardiovascular disease [11, 12], tumors [13–15], diabetes, inflammatory bowel diseases [16], nonalcoholic fatty liver disease [17] and frailty [18]. However, no studies have evaluated the association between ultra-processed food intake and ARHL.

In several observational studies, antioxidants or anti-inflammatory dietary patterns [19, 20] have been found to be inversely related to hearing loss. UPF might be linked to age-related hearing loss in this sense, because the association between UPFs and inflammatory activity has been reported in several studies [21–23]. However, no study has explored the relationship between ARHL and UPFs. This study aimed to examine the associations between ARHL and UPFs based on representative population of the United States.

## Materials and methods

### Study population

We performed this analysis based on the 2015–2017 cycle of National Health and Nutrition Examination Survey (NHANES), which conducted by the National Center for Health Statistics (NCHS). The NHANES is a multistage, stratified probability sample survey that aims to capture a representative sample of the US non-institutionalized civilian population. The NCHS Research Ethics Review Board reviews and approves all NHANES protocols. In this study, data on sociodemographic, lifestyle, audiometry, and nutritional information for persons over the age of 50 were collected from the 2015–2017 period of NHANES. Participants with abnormal otoscopic examination results or tympanogram compliance below 0.3 mL were excluded. Fig. S1 depicted the participants’ screening procedure in this study.

### Dietary assessment

This study employed dietary data from in-person 24-hour dietary recalls completed by trained interviewers fluent in English or Spanish using the validated US Department of Agriculture (USDA) Automated Multiple-Pass Method (AMPM) [24]. Based on Nova food Based on Nova food categorization, UPFs are processed industrial foods that are high in fat, saturated fat, sugar, and salt and do not contain or only contain a little percentage of whole foods. All food items recorded in NHANES 2015–2017 were classified as ultra-processed or non-ultra-processed.

The variables “main food description” and underlying “SR code description” were assessed simultaneously to classify all recorded food items. The phrase " additional food description” was also used in some circumstances. The underlying ingredients (SR codes) were utilized to estimate UPFs energy and gram intakes when meal codes were deemed to be home recipes. Most “frozen meals” or “Lunchables” or food products purchased from a “vending machine” or at a “restaurant fast food/pizza” were classified as ultra-processed foods. Food items that are not frozen, fast-food, or vending machine goods might be deemed home recipes or not. These problems were addressed by taking a cautious approach and presuming those food items were home recipes [25]. The application of the Nova categorization to the NHANES dataset and comprehensive information on how to recognize ultra-processed meals can be found elsewhere [26, 27].

To reflect participants’ UPF consumption, the proportion of UPF in total calorie intake (% UPF) was computed.

### Auditory testing

In the mobile examination center (MEC), all audiometric exam parts were conducted by trained examiners to participants in sound-isolating booths. Participants who couldn’t remove their hearing aids for testing and those who suffered significant ear pain at the time of the exam to be unable to tolerate headphones were eliminated from the audiometry component. A tympanometer was utilized to acquire tympanogram data, and an otoscope (model 25,020) from Welch Allyn was used to examine the ears. Participants’ hearing thresholds were tested at seven frequencies in both ears (500, 1000, 2000, 3000, 4000, 6000, and 8000 Hz).

The pure tone average (PTA) of the hearing thresholds at 0.5 kHz, 1 kHz, and 2 kHz was used to determine the low-frequency pure-tone hearing average (LFPTA). The pure tone average (PTA) of the hearing thresholds at 3k, 4k, 6k, and 8k Hz was used to calculate the high-frequency pure-tone hearing average (HFPTA). Between the two ears, the PTA in the worse ear is chosen.

When either ear meets the criteria for pure-tone hearing threshold > 25 dB [1], normal otoscopic examination results, and compliance of tympanogram > 0.3 mL, sensorineural hearing loss (SNHL) is inferred.

### Covariates

All of the following variables were thought to be possible confounders: Biological sex, age, education level (less than high school, high school graduate or GED, some college or AA, college graduate or more), family poverty-income ratio (PIR), race (non-Hispanic black, non-Hispanic white, or other) and BMI. Smoking status (never, past, or current smoker), energy intake, work-related noise exposure (a response to the question “Have you ever been exposed to loud noise at work?“), firearm exposure (a response to the question “Ever used firearms?“), recreational noise exposure (a response to the question “Have you ever been exposed to loud noise while not at work?“), self-reported chronic diseases (diabetes, hypertension) and ototoxic medication use were also factors to consider (anticancer drugs, aminoglycosides, nonsteroidal anti-inflammatory drugs, and diuretics [28]).

### Statistical analysis

The cycles 2015/2016 and 2017/2018 were merged. According to NHANES tutorials, 4-year sample weights were computed by dividing the 2-year sampling weight by half (WTMEC2YR). For categorical and continuous data, we utilized weighted per-centages or means, accordingly. The odds ratios (ORs) and 95% confidence intervals (CIs) The association between the risk of hearing loss and UPF intake were examined based on the multiple adjusted logistic regression model, using the lowest quartile as a reference. Model 1 was adjusted for age, Biological sex and race. Model 2 was adjusted for age, Biological sex, race, educational level, family poverty-income ratio, BMI, smoking and energy intake. Model 3 was adjusted for all the covariables. We also explored statistical interactions and con-ducted subgroup analyses where they were found to be significant. In addition, restricted cubic spline model was used to flexibly model potential nonlinear relations between UPF intake and risk of hearing loss after adjusting for all the covariables and non-linearity was assessed using the Wald test.

In the sensitivity analysis, weighted multivariable linear regression and restricted cubic spline model was used to examine the association between log-transformed PTA and UPF consumption. Furthermore, we have redefined deafness as a condition in which the hearing threshold of the better-hearing side exceeds 20dB. This redefinition allows us to re-examine the relationship between ARHL and the consumption of UPFs. Lastly, our study delves into the relationship between age-related hearing loss and the quintiles of UPF consumption.

The R Project for Statistical Computing (version 4.0.4) was used to conduct all statistical analyses. Restrictive cubic splines were fitted using the “rms” program in R, and weighted logistic regression models were fitted using the “survey” package.

## Results

### Characteristics

The survey-weighted participant characteristics of the U.S. individuals over 50 included in this study are shown in Table 1. Non-Hispanic Whites made up the bulk of participants (77.2%), as did women (57.8%). The UPF consumption ranged from 0 to 100%: the first quartile (%UPF range from 0 to 32.6%), the second quartile (%UPF range from 32.6 to 51.5%), the third quartile (%UPF range from 51.5 to 71.0%) and the fourth quartile (%UPF range from 71.0 to 100%). Table S1 displays the baseline characteristics of the included individuals stratified by UPF intake quartiles. Participants in the fourth quartile of UPF consumption were more likely to be Non-Hispanic White and less educated than those in the first quartile.

**Table 1:** Characteristics of study population

	Participants
Total (n)	1075
Age(mean ± SD)	61.92 (8.67)
Biological sex:	
Men	469 (42.2)
Women	606 (57.8)
Race:	
Non-Hispanic Black	210 (8.4)
Non-Hispanic White	454 (77.2)
Other	411 (14.4)
Education:	
Less than high school	219 (9.7)
High school graduate or GED	264 (22.5)
Some college or AA	329 (32.8)
College graduate or more	263 (35.1)
Cigarette Smoking:	
Never Smoker	540 (51.1)
Former Smoker	351 (34.0)
Current Smoker	184 (14.9)
Diabetes:	
Yes	276 (20.7)
NO	799 (79.3)
Hypertension:	
Yes	563 (47.0)
NO	512 (53.0)
Use of ototoxic drug:	
NO	963 (90.7)
Yes	112 (9.3)
Occupational noise exposure, n (%)	
Yes	389 (34.5)
NO	686 (65.5)
Firearm noise exposure, n (%)	
Yes	463 (53.4)
NO	612 (46.6)
Recreational noise exposure, n (%)	
Yes	116 (12.9)
NO	959 (87.1)
HFHL:	
NO	313 (30.0)
Yes	762 (70.0)
LFHL:	
NO	793 (78.1)
YES	282 (21.9)
UPF consumption (mean ± SD)	0.47 (0.23)
PIR (mean ± SD)	3.37 (1.58)
BMI (mean ± SD)	30.12 (6.97)
Energy intakes (mean ± SD)	2074.82 (842.17)

BMI, body mass index; SD, the standard deviation

### Association between UPF consumption and hearing loss

**Fig. 1:**
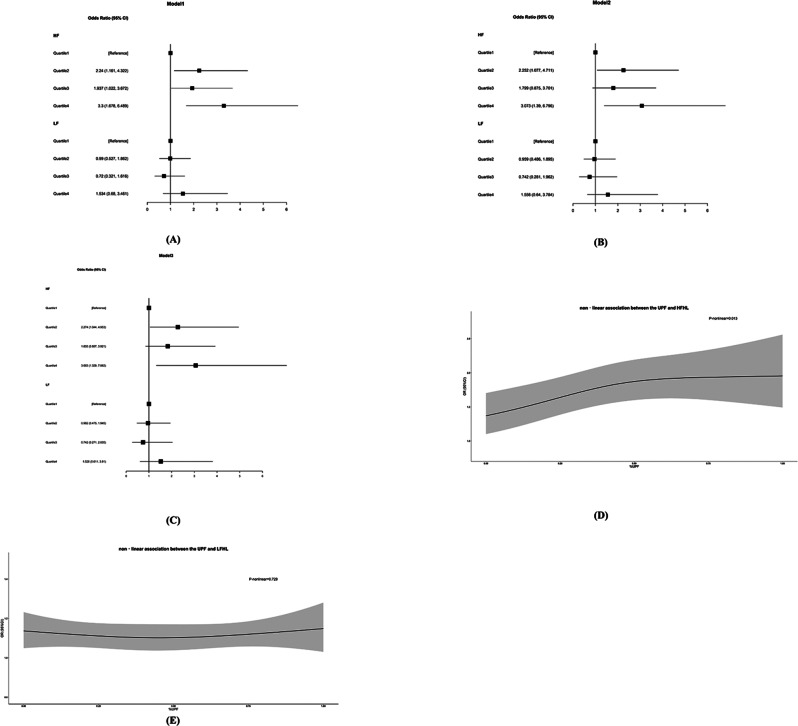
Association between UPF consumption and risk of hearing loss: (**A**-**C**) Forest plot of the associations between the UPF consumption quartiles and risk of hearing loss, the 95% confidence interval (CI) was used to determine if an association was significant, model 1 (**A**) was adjusted for age, Biological sex and race. model 2 (**B**) was adjusted for age, Biological sex, race, educational level, family poverty-income ratio, BMI, smoking, work-related noise exposure, firearm exposure, recreational noise exposure and energy intake. Model 3 (**C**) was adjusted for all the covariables.; (**D**-**E**) Restricted cubic spline regression revealed a nonlinear relationship between UPF consumption and risk of hearing loss after adjusting for all the covariables. P-nonlinear values were from the Wald test, and the black line and gray region indicate the estimated values and their related 95% confidence intervals

We first performed weighted multivariate logistic regression analysis to explored possible associations between UPF consumption and hearing loss. Significant association between UPF consumption and risk of high-frequency hearing loss (HFHL) was observed in different model (Fig. 1A and C). We found that compared with the first quartile of UPF consumption, the ORs of UPF consumption and HFHL for the second, third, and fourth quartiles were 2.274 (1.044, 4.953), 1.833 (0.857, 3.921) and 3.063 (1.329, 7.062) after adjusting for all the covariables (Fig. 1C, Table S2). Significant nonlinearity association between UPF consumption and HFHL was also found using weighted restricted cubic spline regression after adjusting for all the covariables (*p* = 0.013), The risk of HFHL gradually increased with the proportion of UPF consumption and leveled off when UPF consumption reached 50% (Fig. 1D). However, UPF consumption were not found to be associated with Low-frequency hearing loss (LFHL) in different logistic regression models and restricted cubic spline regressions (Fig. 1A, C and E, Table S3).

### Subgroup analysis

**Fig. 2:**
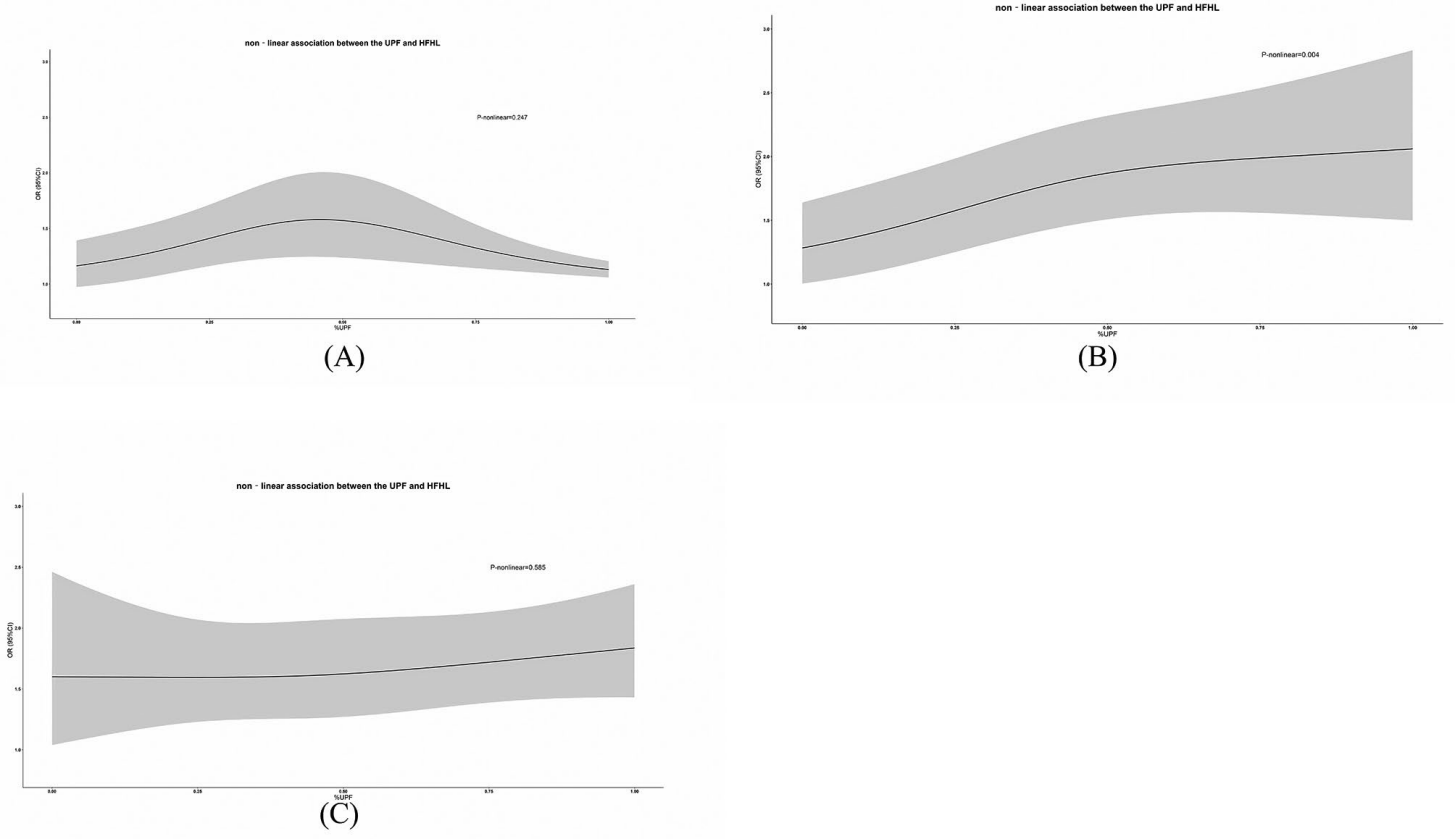
Subgroup analyses: (**A**-**C**) Restricted cubic spline regression in different subgroups; The calculated values and their related 95% CIs are represented by the black line and gray area; P-nonlinear values are from the Wald test: (**A**) Non-Hispanic Black; (**B**) Non-Hispanic White; (**C**) Other

We analyzed the interaction effects of age, sex, race and BMI on the association between UPF consumption and HL by weighted logistic regression model and weighted restricted cubic spline regression model. Interaction effects of age, sex and BMI on the association between UPF consumption and HL were not found. Significant nonlinear interaction effects of race on the association between UPF consumption and HFHL were observed (Table 2). Subgroup analysis by race showed that the nonlinear association between ultra-processed food consumption and HFHL was more significant in non-Hispanic whites (*p* = 0.004) and the shape of the curve was similar to that of the whole population (Fig. 2B). Interestingly, inverted U-shaped curve was presented in Non-Hispanic Black although it was non- significant (*p* = 0.247) (Fig. 2A). Ultra-processed food intake did not appear to be strongly associated with HFHL in older adults of other ethnicities (Fig. 2C).

**Table 2:** P values of interaction terms in different models

	Age	Biological sex	Race	BMI
Quartiles terms				
HFHL	0.52	0.77	0.16	0.08
LFHL	0.7	0.41	0.2	0.65
Nonlinear terms				
HFHL	0.88	0.43	**0.02**	0.19
LFHL	0.34	0.11	0.76	0.85

The bold font represents a statistically significant P-value

### Sensitivity analyses

#### Association between log-transformed PTA and UPF

**Fig. 3:**
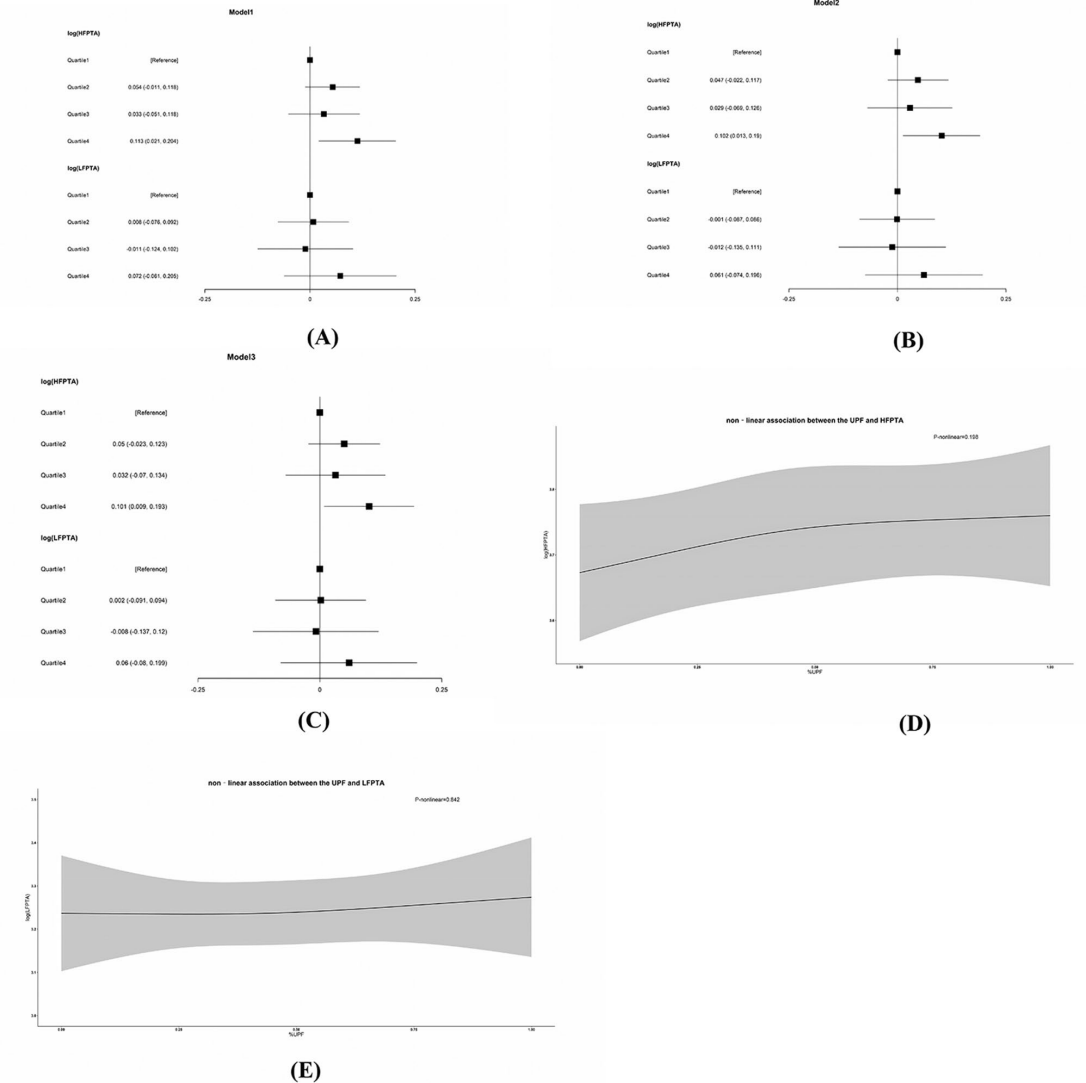
Association between UPF consumption and PTA: (**A**-**C**) Forest plot of the associations between the UPF consumption quartiles and risk of HL, the 95% confidence interval (CI) was used to determine if an association was significant, Model 1 (**A**) was adjusted for age, Biological sex and race. model 2 (**B**) was adjusted for age, Biological sex, race, educational level, family poverty-income ratio, BMI, smoking, work-related noise exposure, firearm exposure, recreational noise exposure and energy intake. Model 3 (**C**) was adjusted for all the covariables. (**D**-**E**) Restricted cubic spline regression revealed a nonlinear relationship between UPF consumption and PTA after adjusting for all the covariables. P-nonlinear values were from the Wald test, and the black line and gray region indicate the estimated values and their related 95% confidence intervals

We examined the association between log-transformed PTA and UPF consumption by weighted multivariable linear regression and weighted restricted cubic spline regression. Compared with the first quartile of UPF consumption, β value of fourth quartiles were 0.101 (0.009, 0.193) after adjusting for all the covariables (Fig. 3C). Similar shape of the curve was also observed in weighted restricted cubic spline regression although it is non- significant (*p* = 0.198) (Fig. 3D).The association between UPF consumption and LFPTA was also not found in weighted multivariable linear regression and weighted restricted cubic spline regression (Fig. 3A, C and E).

After adopting the new definition of deafness (where the hearing threshold of the better-hearing ear exceeds 20dB), we found that, even after adjusting for all covariates, the consumption of UPFs remains positively associated with the incidence of high-frequency age-related hearing loss. Compared to the first quartile of UPF consumption, the fourth quartile demonstrated an OR and 95% CI of 2.326 (1.016, 5.325) for the risk of high-frequency hearing loss (Fig. S2).

Utilizing quintiles of UPFs consumption, and after adjusting for all covariates, we observed that compared to the first quintile, the second, third, fourth, and fifth quintiles of UPF consumption are associated with increasing ORs and 95% Cis for the risk of high-frequency hearing loss. Specifically, these ORs and 95% CIs are 1.64 (0.655, 4.102), 2.759 (1.188, 6.409), 1.752 (0.841, 3.65), and 3.044 (1.239, 7.477) for the second, third, fourth, and fifth quintiles respectively (Fig. S3).

## Discussion

In this study, we discovered that among US individuals aged 50 and over, UPF intake was significantly correlated with HFHL. After controlling for all covariables, individuals in the fourth quartile of UPF consumption had a 3 times higher chance of developing HFHL than individuals in the first quartile of UPF consumption. Consistent results were also obtained in sensitivity analysis. In comparison to the first quartile, the differences between the second and fourth quartiles hold more statistical significance. By incorporating restrictive cubic spline regression, we suggest that these findings indicate a more fitting curve model for the relationship between the intake of highly processed foods and the risk of hearing loss in middle-aged and elderly individuals. However, we still lack a complete understanding of this nonlinear relationship, emphasizing the need for future studies with larger sample sizes to further explore this phenomenon. We also found that the association was more significant in non-Hispanic whites.

Given our inability to definitively ascertain whether the consumption of UPFs leads to an increased risk of asymmetrical hearing loss, this study redefined deafness as a threshold greater than 25dB in the poorer-hearing ear. In our sensitivity analysis, we also employed this updated definition of deafness. We discovered that, even with the revised definition, the consumption of UPFs remains significantly associated with the risk of high-frequency hearing loss in middle-aged and older adults (see Fig. S3). However, the significance was weaker, which may suggest that UPF consumption could potentially increase the risk of asymmetrical hearing loss in this population.

Interestingly, in this study, we observed that the relationship between UPFs consumption and age-related hearing loss appears to follow a complex curvilinear association. Compared to the first quartile, the risk of high-frequency hearing loss in middle-aged and older adults was more significant in the second and fourth quartiles. In subsequent sensitivity analyses using quintiles of UPF consumption, this complex curvilinear relationship persisted. The underlying reasons for this phenomenon are currently unclear, necessitating further exploration in future research.

Several previous studies have suggested that healthy diets were related with lower risk of hearing loss [7, 8, 19, 20], which is consistent with results from the present study. However, prior investigators have often concentrated on nutrients, foods, or dietary habits. Due to improvements in food processing and technology over the past few decades, the world’s food systems have undergone significant changes, industrially processed and prepared food products are replacing traditional diets that stress whole or little processed foods, home cooking, and food preparation. Instead of focusing on individual nutrients or particular food items, categorizing foods and beverages according to their level of food processing may offer unique insight into the dietary factors that increase the risk of ARHL by defining a class of foods with poor nutritional quality [29]. To the best of our knowledge, this study is the first one aiming to study the associations be-tween UPF and ARHL.

There are a number of ways that UPF consumption may increase risk of ARHL. High consumption of UPFs will lead to lower intake of non-ultra-processed foods [30], such as fresh fruits and vegetables, which will ultimately result in a poor diet quality, which has been linked to a higher risk of hearing loss [31]. In addition, chemicals that some may have harmful effects on hearing may migrate from packing materials to food ingredients. According to a cross-sectional study, UPFs consumption may expose people to more phthalates [32], which have been found a significant association with risk of hearing loss [33]. The negative impact of UPFs on the gut microbiome, which is considered a master regulator of immune homeostasis [34], may also increase the likelihood of developing ARHL. Probiotics in gut may be reduced as a result of the poor nutritional content of UPF [35]. Disturbed gut microbiome could lead to intestinal metabolism disorder and inflammatory bowel disease. A leaky gut caused by a pro-inflammatory gut environment might allow infections and their metabolites to travel via the circulation to distant organs including the brain and the cochlea [36, 37], which in turn leads to hearing impairment. In addition, the results of this study showed that UPF intake had a more significant effect on HFHL. It is generally believed that age-related hearing loss first occurs in the high frequency hearing threshold [1, 2]. The high frequency hearing threshold is more likely to be impaired in middle-aged and elderly people, which may be the reason why UPF is more significantly associated with high frequency hearing loss.

It’s interesting that our study discovered that non-Hispanic whites had higher than average HFHL risks. In actuality, the risk of ARHL varies depending on race. Numerous large population-based cohort epidemiologic studies have found that black individuals had lower rates of hearing loss than white people, with the likelihood of hearing loss being generally 40–60% lower in black people [38]. It was also found that darker-skinned Hispanics also had significantly better hearing than lighter-skinned Hispanics [39]. Melanocyte function may be the underlying mechanism causing the connection between race and ARHL. It has been suggested that the melanin produced by strial melanocytes in the cochlea acts as a metal chelator or a free radical scavenger to provide protection [40].

This study has several advantages. First, the sizeable and nationally representative NHANES sample contributes to accurate results. Additionally, in order to minimize the interference of covariates as much as feasible, we made adjustments for a variety of probable relevant aspects of ARHL in regression models. Second, in our study, hearing loss was assessed using objectively measured audiometry tests, which was regarded as the gold standard [41] We must acknowledge, however, that this study has certain limitations. Firstly, due to the cross-sectional nature of our findings, causal as-sociations between UPFs consumption and ARHL cannot be established. Secondly, there are numerous and diverse food processing techniques, making it challenging to gauge the degree of processing. For some foods, it is challenging to categorize them precisely. Thirdly, the accuracy of a 24-h dietary recall interview is heavily reliant on the participants’ memory and may not accurately reflect individuals’ daily diet in a single 24-h dietary recall. Fourthly, this study did not consider the impact of UPF on asymmetrical hearing loss. Finally, we found that the relationship between the consumption of UPFs and the risk of high-frequency hearing loss in middle-aged and older adults may follow a complex curvilinear pattern. However, the reasons behind this curvilinear relationship remain unexplained, necessitating more in-depth future research to explore this phenomenon.

In conclusion, this study discovered an association between UPF intake and the incidence of HFHL, which was more significant in non-Hispanic whites. To support our findings, additional longitudinal studies with dietary data that reflects the con-temporary food supply are required.

### Electronic supplementary material

Below is the link to the electronic supplementary material.

Supplementary Material 1

Supplementary Material 2

Supplementary Material 3

Supplementary Material 4

Supplementary Material 5

Supplementary Material 6

## Data Availability

All data for this study are available on the NHANES website: https://www.cdc.gov/nchs/nhanes/index.htm? CDC_AA_refVal=https%3 A%2 F%2Fwww.cdc.gov%2Fnchs%2Fnhanes.htm.
