# Supplementary material for: The association between ultra-processed food intake and age-related hearing loss: a cross-sectional study
Source: BMC Geriatr. 2024 May 23;24:450. doi: 10.1186/s12877-024-04935-0 (PMC11118724; doi:10.1186/s12877-024-04935-0)
Supplement: Supplementary file 1 — Supplementary Material 1 [file 12877_2024_4935_MOESM1_ESM.docx]

|  | Quartile1 | Quartile2 | Quartile3 | Quartile4 |
| --- | --- | --- | --- | --- |
| Total (n) | 339 | 318 | 251 | 167 |
| Age (mean ±SD) | 62.48 (8.37) | 62.36(9.20) | 61.51(8.29) | 60.85 (8.73) |
| Gender： |  |  |  |  |
| Men | 139 (36.4) | 143(46.9) | 114(45.2) | 73 (37.2) |
| Women | 200 (63.6) | 175 (53.1) | 137(54.8) | 94 (62.8) |
| Race: |  |  |  |  |
| Non-Hispanic Black | 61 (9.2) | 62 (8.7) | 51 (7.2) | 36 (8.5) |
| Non-Hispanic White | 99 (67.1) | 135 (77.5) | 132 (83.2) | 88 (82.6) |
| Other | 179 (23.7) | 121 (13.7) | 68 (9.5) | 43 (8.9) |
| Education |  |  |  |  |
| Less than high school | 87 (11.5) | 51 (9.5) | 43 (7.4) | 38 (11.5) |
| High school graduate or GED | 72 (18.8) | 82 (19.6) | 64 (24.7) | 46 (30.2) |
| Some college or AA | 82 (28.3) | 106 (35.7) | 83 (31.6) | 58 (36.7) |
| College graduate or more | 98 (41.4) | 79 (35.2) | 61 (36.3) | 25 (21.6) |
| Cigarette Smoking |  |  |  |  |
| NO | 201 (55.1) | 163 (57.1) | 114(44.8) | 62 (44.0) |
| Yes | 93 (32.4) | 112 (31.1) | 88 (38.5) | 58 (34.2) |
|  | 45 (12.5) | 43 (11.7) | 49 (16.7) | 47 (21.7) |
| Diabetes |  |  |  |  |
| Yes | 98 (22.0) | 86 (20.3) | 49 (18.2) | 43 (24.1) |
| NO | 241 (78.0) | 232 (79.7) | 202 (81.8) | 124 (75.9) |
| Hypertension |  |  |  |  |
| Yes | 175 (43.1) | 167 (47.4) | 132 (49.6) | 89 (48.4) |
| NO | 164 (56.9) | 151 (52.6) | 119 (50.4) | 78 (51.6) |
| Use of ototoxic drug |  |  |  |  |
| NO | 310 (92.5) | 289 (91.4) | 219 (89.6) | 145 (88.4) |
| Yes | 29 (7.5) | 29 (8.6) | 32 (10.4) | 22 (11.6) |
| HFHL |  |  |  |  |
| NO | 113 (38.6) | 84 (26.1) | 77 (29.2) | 39 (24.2) |
| Yes | 226 (61.4) | 234 (73.9) | 174 (70.8) | 128 (75.8) |
| LFHL |  |  |  |  |
| NO | 251 (76.4) | 229 (76.7) | 199 (83.3) | 114 (73.9) |
| YES | 88 (23.6) | 89 (23.3) | 52 (16.7) | 53 (26.1) |
| UPF consumption (mean ±SD) | 0.18 (0.09) | 0.42 (0.06) | 0.61 (0.05) | 0.82 (0.08) |
| PIR (mean ±SD) | 3.41 (1.64) | 3.35 (1.59) | 3.47 (1.52) | 3.19 (1.54) |
| BMI (mean ±SD) | 28.78 (6.10) | 29.70 (6.35) | 30.85 (7.04) | 31.91 (8.73) |
| energy intakes (mean ±SD) | 1872.21 (718.68) | 2140.77 (885.50) | 2175.76 (856.32) | 2107.85 (873.12) |

Table S1 Characteristic of participants by UPF intake quartiles

**Abbreviations:** BMI, body mass index; SD, the standard deviation.
