# Supplementary material for: The association between ultra-processed food intake and age-related hearing loss: a cross-sectional study
Source: BMC Geriatr. 2024 May 23;24:450. doi: 10.1186/s12877-024-04935-0 (PMC11118724; doi:10.1186/s12877-024-04935-0)
Supplement: Supplementary file 2 — Supplementary Material 2 [file 12877_2024_4935_MOESM2_ESM.docx]

|  | Model 1 | | Model 2 | | Model 3 | |
| --- | --- | --- | --- | --- | --- | --- |
|  | p | OR | p | OR | p | OR |
| Quartil1 | Reference | Reference | Reference | Reference | Reference | Reference |
| Quartil2 | <0.001 | 2.24 | 0.033 | 2.252 | 0.041 | 2.274 |
| Quartil3 | <0.001 | 1.937 | 0.100 | 1.799 | 0.104 | 1.839 |
| Quartil4 | <0.001 | 3.3 | 0.009 | 3.073 | 0.014 | 3.063 |

**Table S2 Odds ratio and p values in HF models**
