# Supplementary material for: The association between ultra-processed food intake and age-related hearing loss: a cross-sectional study
Source: BMC Geriatr. 2024 May 23;24:450. doi: 10.1186/s12877-024-04935-0 (PMC11118724; doi:10.1186/s12877-024-04935-0)
Supplement: Supplementary file 3 — Supplementary Material 3 [file 12877_2024_4935_MOESM3_ESM.docx]

**Table S3 Odds ratio and p values in LF models**

|  | Model 1 | | Model 2 | | Model 3 | |
| --- | --- | --- | --- | --- | --- | --- |
|  | p | OR | p | OR | p | OR |
| Quartil1 | Reference | Reference | Reference | Reference | Reference | Reference |
| Quartil2 | 0.972 | 0.990 | 0.893 | 0.959 | 0.901 | 0.962 |
| Quartil3 | 0.401 | 0.721 | 0.512 | 0.742 | 0.522 | 0.742 |
| Quartil4 | 0.282 | 1.534 | 0.292 | 1.556 | 0.323 | 1.526 |
