# Supplementary figures and images for: The association between ultra-processed food intake and age-related hearing loss: a cross-sectional study
Source: BMC Geriatr. 2024 May 23;24:450. doi: 10.1186/s12877-024-04935-0 (PMC11118724; doi:10.1186/s12877-024-04935-0)

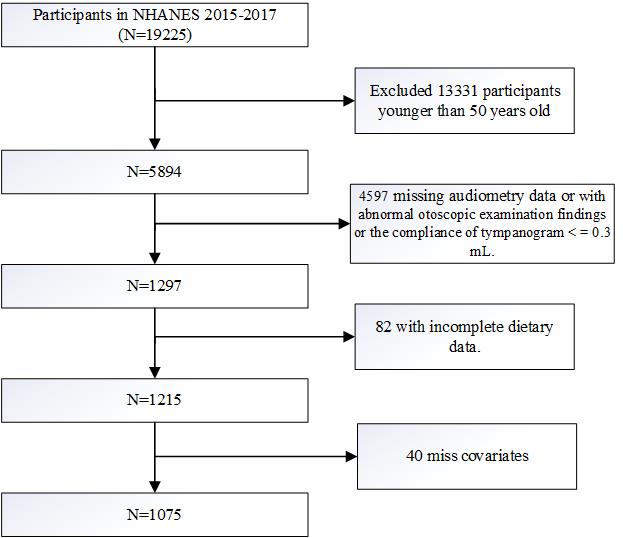


**Figure S1** The participants' screening procedure in this study

Supplement: Supplementary file 4 — Supplementary Material 4 [file 12877_2024_4935_MOESM4_ESM.docx]
