# Supplementary material for: The association between ultra-processed food intake and age-related hearing loss: a cross-sectional study
Source: BMC Geriatr. 2024 May 23;24:450. doi: 10.1186/s12877-024-04935-0 (PMC11118724; doi:10.1186/s12877-024-04935-0)
Supplement: Supplementary file 5 — Supplementary Material 5 [file 12877_2024_4935_MOESM5_ESM.docx]

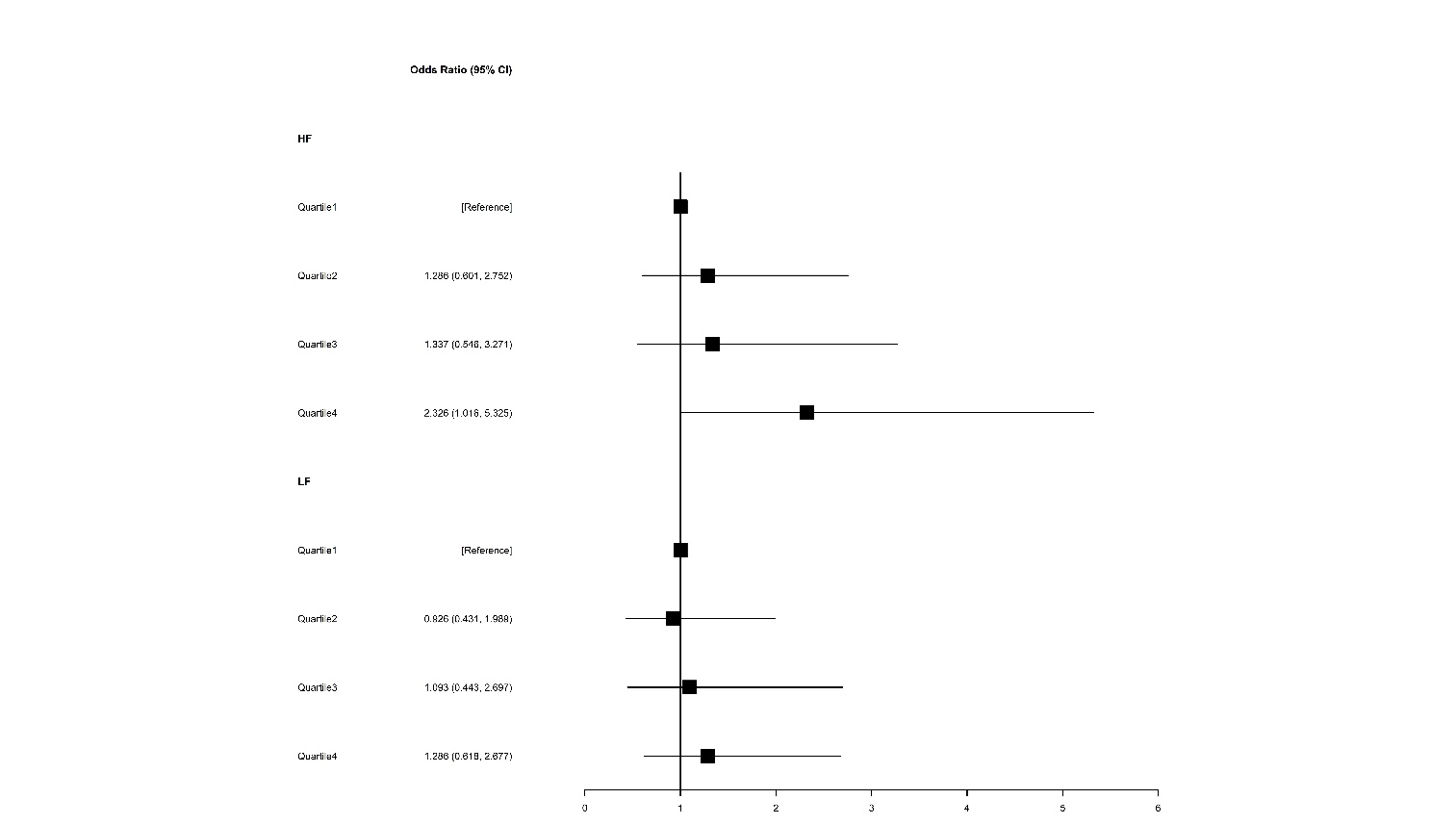


**Figure S2:** Association between the risk of hearing loss under the new definition (hearing threshold greater than 20dB in the better-hearing ear) and quartiles of Ultra-Processed Food (UPF) consumption, with all models adjusted for all covariates.
