# Supplementary material for: The association between ultra-processed food intake and age-related hearing loss: a cross-sectional study
Source: BMC Geriatr. 2024 May 23;24:450. doi: 10.1186/s12877-024-04935-0 (PMC11118724; doi:10.1186/s12877-024-04935-0)
Supplement: Supplementary file 6 — Supplementary Material 6 [file 12877_2024_4935_MOESM6_ESM.docx]

**Figure S3:** Relationship between the risk of hearing loss in middle-aged and older adults and quintiles of Ultra-Processed Food (UPF) consumption, with all models adjusted for all covariates.
